# Supplementary material for: Evaluation of the 8th Edition AJCC Staging System for the Clinical Staging of Pancreatic Cancer
Source: Cancers (Basel). 2022 Sep 26;14(19):4672. doi: 10.3390/cancers14194672 (PMC9563770; doi:10.3390/cancers14194672)
Supplement: Supplementary file 1 [file cancers-14-04672-s001.zip › cancers-1897177-supplementary.pdf]

Supplementary Materials

Evaluation of the 8th Edition AJCC Staging System for the Clinical Staging of Pancreatic Cancer

Supplementary material S1. Detailed information of neoadjuvant and adjuvant therapy.

**Neoadjuvant therapy (n=100)**	
CCRT with 5-FU	49 (49.0%)
CCRT with gemcitabine	13 (13.0%)
FOLFIRINOX	20 (20.0%)
Gemcitabine based CTx†	10 (10.0%)
Other CTx*	8 (8.0%)
**Adjuvant therapy (n=129)**	
Gemcitabine monotherapy	97 (75.2%)
Gemcitabine based combination CTx∫	4 (3.1%)
5-FU monotherapy	11 (8.5%)
5-FU based combination CTx∫∫	17 (13.2%)

Abbreviations: CCRT, concurrent chemoradiotherapy; 5-FU, 5-fluorouracil; CTx, chemotherapy. † Includes eight gemcitabine + erlotinib, one gemcitabine + nab-paclitaxel, and one gemcitabine therapy. * Includes three capecitabine, three tegafur/uracil, and two 5-fluorouracil therapy. ∫ Includes three gemcitabine + erlotinib, and one gemcitabine + capecitabine therapy. ∫∫ Includes twelve 5-fluorouracil + etoposide + cisplatin, and five 5-fluorouracil + cisplatin therapy.

Supplementary material S2. Kaplan–Meier estimates of overall survival of stage groups according to the AJCC staging system, 8th edition in patient with confirmed adenocarcinoma (**A**) Comparison of stages (from I to IV), (**B**) Comparison of substages (from IA to IIB).

(A)

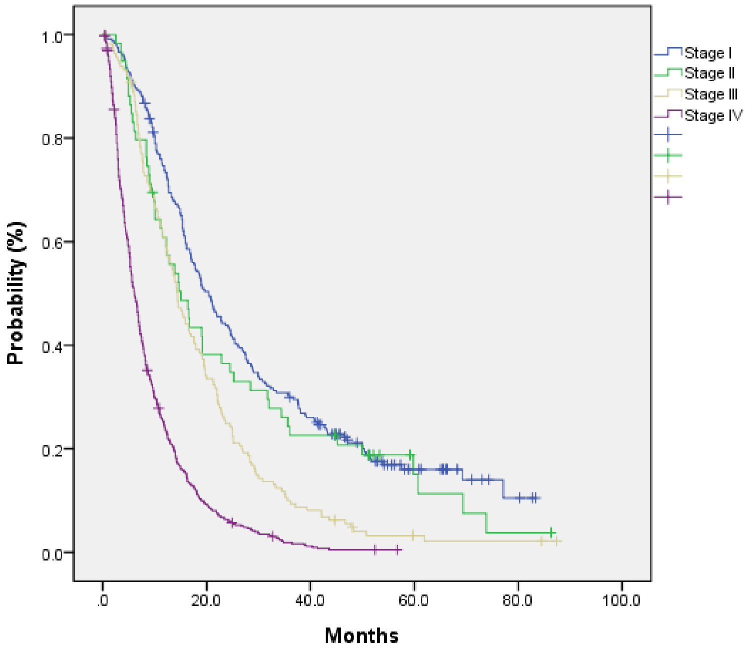

Stage group	Median OS, months (95% CI)
I (n=234)	20.4 (17.3–23.5)
II (n=59)	15.1 (11.0–19.2)
III (n=163)	14.4 (12.5–16.3)
IV (n=486)	6.1 (5.4–6.8)

P	I	II	III
I			
II	0.225		
III	**<0.001**	**0.026**	
IV	**<0.001**	**<0.001**	**<0.001**

(B)

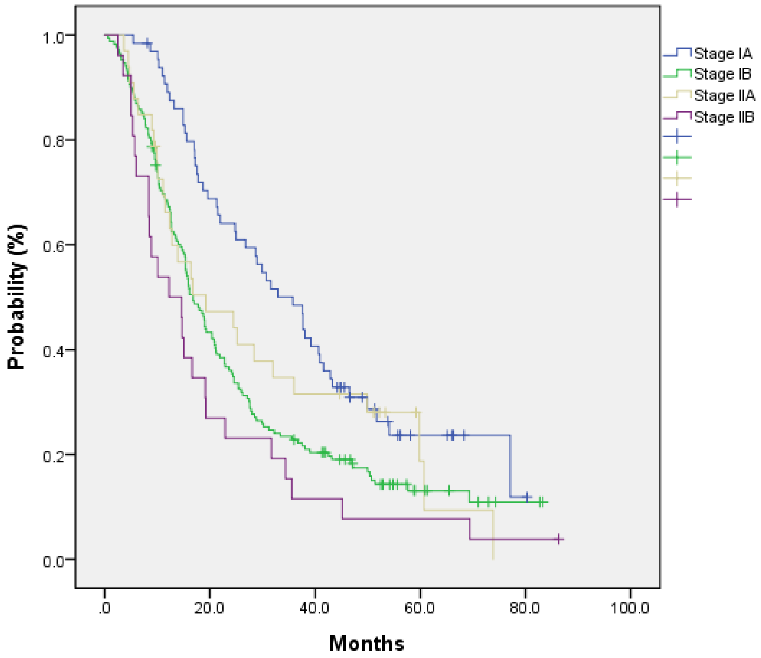

Stage group	Median OS, months (95% CI)
IA (n=65)	35.8 (27.3–44.3)
IB (n=169)	16.8 (14.3–19.3)
IIA (n=33)	19.2 (4.7–33.7)
IIB (n=26)	12.2 (5.0–19.4)

P	IA	IB	IIA
IA			
IB	**<0.001**		
IIA	0.128	0.357	
IIB	**<0.001**	0.109	0.095

Supplementary material S3. Overall survival of non-metastatic pancreatic adenocarcinoma patients according to the AJCC staging system, 8th edition stratified by N staging.

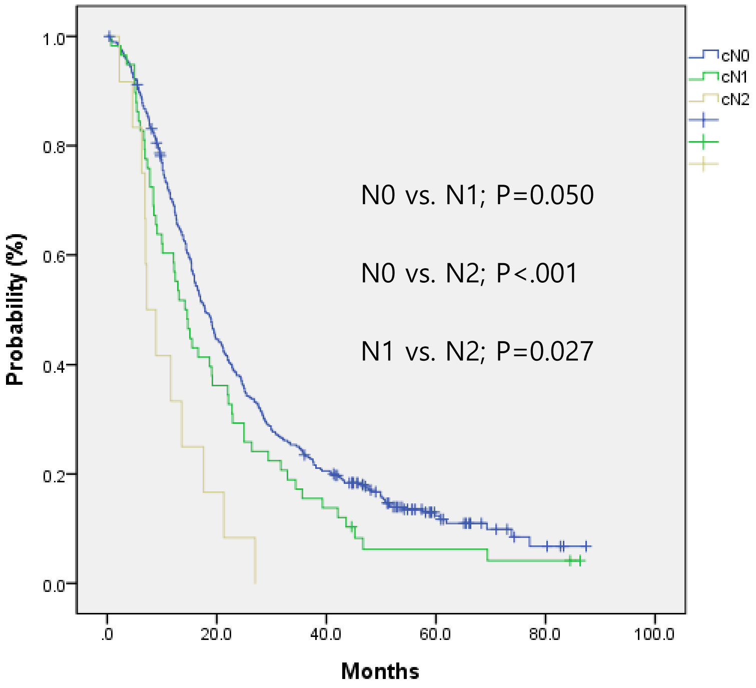

N stage	Median OS, months (95% CI)
N0 (n=386)	17.9 (16.0–19.8)
N1 (n=58)	14.2 (10.8–17.6)
N2 (n=12)	7.2 (4.0–10.4)
